# Genome and proteome analysis of 7-7-1, a flagellotropic phage infecting *Agrobacterium* sp H13-3

**DOI:** 10.1186/1743-422X-9-102

**Published:** 2012-05-31

**Authors:** Andrew M Kropinski, An Van den Bossche, Rob Lavigne, Jean-Paul Noben, Patrick Babinger, Rüdiger Schmitt

**Affiliations:** 1 Laboratory for Foodborne Zoonoses, Public Health Agency of Canada, Guelph, ON, NIG 3W4, Canada; 2 Department of Molecular & Cellular Biology, University of Guelph, Guelph, NIG 2W1, ON, Canada; 3 Division of Gene Technology, Katholieke Universiteit Leuven, Kasteelpark Arenberg 21, 3001, Heverlee, Belgium; 4 Biomedical Research Institute and Transnational University Limburg, School of Life Sciences, Hasselt University, Diepenbeek, Belgium; 5 Institut für Biophysik und physikalische Biochemie, Universität Regensburg, D-93040, Regensburg, Germany; 6 Institut für Biochemie, Genetik und Mikrobiologie, Universität Regensburg, D-93040, Regensburg, Germany

**Keywords:** *Agrobacterium*, Phage evolution, Phage ecology, Genome, Proteome, Complex flagellum, Bioinformatics, Posttranslational modification

## Abstract

**Background:**

The flagellotropic phage 7-7-1 infects motile cells of *Agrobacterium* sp H13-3 by attaching to and traveling along the rotating flagellar filament to the secondary receptor at the base, where it injects its DNA into the host cell. Here we describe the complete genomic sequence of 69,391 base pairs of this unusual bacteriophage.

**Methods:**

The sequence of the 7-7-1 genome was determined by pyro(454)sequencing to a coverage of 378-fold. It was annotated using MyRAST and a variety of internet resources. The structural proteome was analyzed by SDS-PAGE coupled electrospray ionization-tandem mass spectrometry (MS/MS).

**Results:**

Sequence annotation and a structural proteome analysis revealed 127 open reading frames, 84 of which are unique. In six cases 7-7-1 proteins showed sequence similarity to proteins from the virulent *Burkholderia* myovirus BcepB1A. Unique features of the 7-7-1 genome are the physical separation of the genes encoding the small (orf100) and large (orf112) subunits of the DNA packaging complex and the apparent lack of a holin-lysin cassette. Proteomic analysis revealed the presence of 24 structural proteins, five of which were identified as baseplate (orf7), putative tail fibre (orf102), portal (orf113), major capsid (orf115) and tail sheath (orf126) proteins. In the latter case, the N-terminus was removed during capsid maturation, probably by a putative prohead protease (orf114).

## Background

Bacteriophage 7-7-1 is known to infect motile cells of *Agrobacterium* sp H13-3 (formerly *Rhizobium lupini*[1]), and as such is termed flagellotropic. Using electron microscopy, Lotz et al. [2] demonstrated translocation of phage 7-7-1 along flagellar filaments. Filament associated phage particles initially possess DNA-filled heads, which are subsequently found emptied when attached to the phage receptor at the flagellar base. This bimodal mechanism of adsorption dramatically increases the chance for finding the receptor at the cell surface, because (i) swimming bacteria with their flagella spread out act as a five- to 10-fold expanded target for the phage and, (ii) once attached, phage particles are directed to the receptor by a one-dimensional walk along the flagellum (instead of a random ‘search’ by three-dimensional diffusion). In no case has the process of phage translocation along the flagellum been visualized. Based on circumstantial evidence, Samuel et al. [3] have estimated that the flagellotropic phage *χ* of *Salmonella* needs < 1 s to reach the flagellar base. These authors have also provided evidence for a ‘nut and bolt’ mechanism by which phage *χ* moves along the filament. They argue that the long tail fiber fits the right-handed grooves between helical rows of flagellin subunits and that the counter-clockwise (CCW) rotation of the flagellum forces the phage to follow the grooves as a nut follows the threads of a bolt.

**Figure 1:**
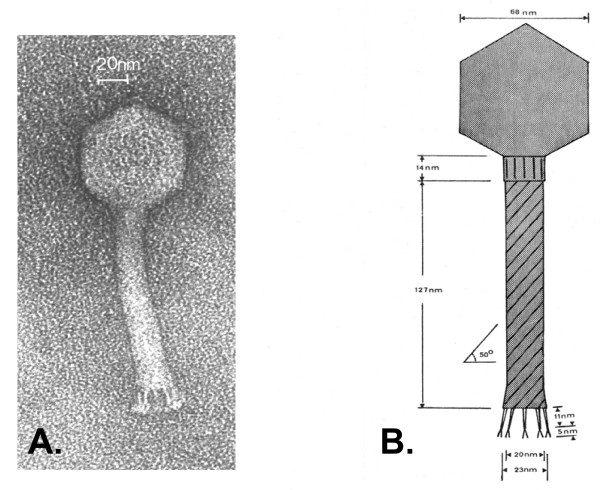
**High-resolution electron micrograph (A) and scale diagram (B) of bacteriophage 7-7-1. A 14-nm collar connects the icosahedral head with the contractile tail that exhibits a surface structure of helical rows running at an angle of 50°.** Five 16-nm tail fibers with splayed tips probably lead the phage along the flagellar filament to the cell surface, where they act as specific adsorption organelles that attach the phage to its final receptor. Details of the tail fine structure were uncovered by optical diffraction [8] of highly resolved electron micrographs.

However, such conditions are not met by the ‘complex’ flagella of *Agrobacterium* sp H13-3. In fact, complex filaments exhibit a prominent pattern of right-handed helical ridges and grooves recommending itself as convenient ‘threads’, but the sense of flagellar rotation is exclusively clockwise (CW; [4-6]). Hence, ‘nut and bolt’ mechanics would force an attached phage particle to the distal end rather than to the flagellar base. Thus, the observed movement of 7-7-1 to the flagellar base demands a different, yet unknown mode of translocation. Differences between the two flagellotropic phages are also reflected by their distinct morphologies: electron micrographs of phage χ show a single long (200–220 nm) tail fiber wrapped around the ‘plain’ filament of *Salmonella*[7], whereas phage 7-7-1 exhibits five short (16 nm) tail fibers with splayed tips. Figure 1B shows a scale diagram of phage 7-7-1 as deduced from high-resolution electron micrographs (Figure 1A). 

7-7-1 is the first flagellotropic phage shown to infect a soil bacterium driven by the uni-directional CW rotation of its complex flagella, a pattern clearly different from the CCW-CW bias of the plain flagella driving *Salmonella*[9]. This departure from the well-studied enterobacterial paradigm and the rare phage morphology prompted us to analyze the genome and the structural proteome of 7-7-1.

## Results

### Genome

**Figure 2:**
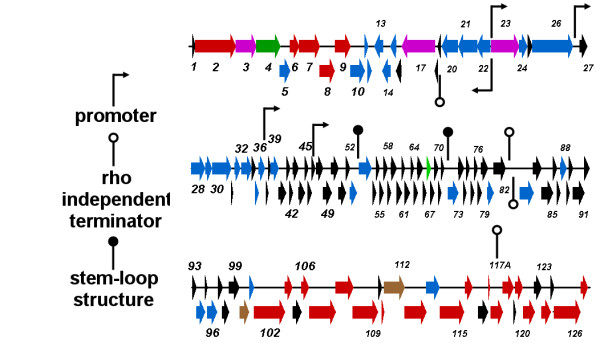
**Genetic map of 7-7-1 showing genes encoding hypothetical proteins in black; conserved hypothetical proteins, blue; structural proteins, red; regulatory proteins, green; DNA and nucleotide metabolism, purple; terminase subunits, brown.** Putative promoters are indicated with black arrows on stalks, while predicted rho-independent terminators are indicated with white circle on stalks, and stem-loop structures are indicated with black circle on stalks.

Electron micrographs of platinum/iridium-stained 7-7-1 DNA revealed mostly linear and a few circular molecules of approximately 25 μm contour lengths (mass of ≅73.5 kb; data not shown) suggesting DNA circularization by cohesive ends. These single-stranded termini are not covered by 454 sequencing. The 454 sequence data revealed that the genome of the phage was 69,391 bp (52.4 mol%G + C). Following automated annotation using MyRAST the genome was manually curated revealing 127 ORFs and no tRNAs. The majority (84, 65.6%) of the ORFs showed no homology to any protein in the current NCBI databases. A minority showed similarity to prophage (28, 21.9%) or phage proteins (16, 12.5%). In the latter case 7-7-1 gp20-26 were collinear to a set of genes from *Burkholderia* phage BcepB1A [10] which is also a virulent myovirus. Phage 7-7-1 displays a number of unique features including the physical separation of the genes encoding the small (orf100) and large (orf112) subunits of the terminase complex. In addition, there is no evidence for a holin-lysin cassette (Figure 2; Additional file 1, Table S1). 

### DNA replication

DNA replication of this phage involves a helicase (orf23) and a polymerase (orf17). The latter shows greatest sequence similarity to the DNA polymerases of *Pseudomonas* phage 73 (YP_001293433) and *Burkholderia* phage BcepGomr (YP_001210246) which are members of the *Siphoviridae*, and *Burkholderia* phage BcepB1A (YP_024903) which, like 7-7-1, is a myovirus. An InterProScan shows it to be a member of the DNA/RNA polymerases superfamily (SUPERFAMILY SSF56672) with the motif located between residues 318 and 480. Two other proteins potentially involved in replication are the products of genes *28* and *33*. Gp28 is a 255 amino acid protein possessing ParB-like nuclease motifs (Pfam PF02195 ParBc; SMART SM00470 ParB-like nuclease domain and SUPERFAMILY [11] SSF110849 ParB/Sulfiredoxin) as well as ParB-like partition TIGRFAMs [12] protein motif TIGR00180 parB_part: ParB-like partition proteins. This type of protein has also been found in myoviruses such as *Burkholderia ambifaria* phage BcepF1 (YP_001039693), *Mycobacterium* phage Pio (AER49600) and enterobacterial phage P1 (AAQ14139). Gp33 contains a N-(deoxy)ribosyltransferase-like superfamily (SUPERFAMILY SSF52309) motif.

### Transcription

Based upon the assumption that the genome circularizes via cohesive termini (not identified), there are two large transcriptional units encompassing orf 22–13 and orf 23–127, 1–12. Since another member of the class α-proteobacteria, *Rhizobium etli*, possesses *rpoD*-dependent promoters which closely resemble the *Escherichia coli* consensus sequence (TTGACA[N15-17]TATAAT) [13] we assumed that this phage might contain recognizable promoters. We identified five potential promoter sequences, including divergent promoters between the two transcription units (Additional file 2, Table S2). In addition four rho-independent terminators were identified and two high ΔG stem-loop structures. Interestingly, no bidirectional terminators were discovered between orf12 and orf13 (Additional file 2, Table S2). No evidence was found as to how transcription is temporally regulated in this virus.

The genome of phage 7-7-1 encodes for two proteins involved in DNA synthesis – a helicase (gp23) and a polymerase (gp17). The polymerase displayed no conserved motifs, and is distantly related to gp43 homologs from cyanomyoviruses. The helicase contained a high scoring (E-value: 1.01e-41) COG1061, DNA or RNA helicases of superfamily II protein motif (SSL2); and, homology to helicases from *Burkholderia* phage BcepB1A [10], and *Vibrio* phages VP16C and VT16T [14].

PSI-BLAST analysis of Gp3 against the NCBI virus database resulted in hits described as tail/DNA circulation protein (*Salmonella* phage ST64B [15], Enterobacteria phage SfV [16], *Pseudomonas* phage DVM-2008, and *Burkholderia* phage KS10 [17]. This protein possesses two protein motifs: COG4228, Mu-like prophage DNA circulation protein, and pfam07157, DNA circulation protein N-terminus (DNA_circ_N) which are conserved protein domains of indeterminate function. Gp4 contains two inconsistent overlapping motifs: COG4379, Mu-like prophage tail protein gpP (E-value: 2.99e-22), and, pfam05954, phage late control gene D protein (Phage_GPD; E-value: 1.76e-17). The homologs include tail proteins from Mu, D108, SfV and ST64B. These results, coupled with the genome location and the observation that Gp3 is a structural protein (see next section), suggest that both of these proteins are involved in the sequence/assembly of the phage tail.

### Virion structural proteins

BLAST analysis revealed several proteins as being involved in phage morphogenesis including baseplate protein (gp7), tail fibre (gp102), portal (gp113), prohead protease (gp114), major capsid (gp115) and tail sheath (gp126). HHpred [18,19] analysis on other proteins in the morphogenesis cassette was used to identify three other proteins - gp5, gp6 and gp10. Gp10 which we had termed a conserved hypothetical membrane protein shows structural similarity (Probability = 91.01; E-value = 0.11) to RCSB Protein Data Bank [20] 3BKH, the lytic transglycosylase (gp144) of *Pseudomonas* phage φKZ which is probably the endolysin for this virus [21]. Gp6 is related (Probability = 83.90; E-value = 0.63) to 2IA7 – a putative tail lysozyme (T4 gp5 analog); while gp5 is a structural analog of 3AJQ, phage P2 protein V which is the tailspike protein (Probability = 96.23; E-value = 0.021) [22].

### Proteomics

**Figure 3:**
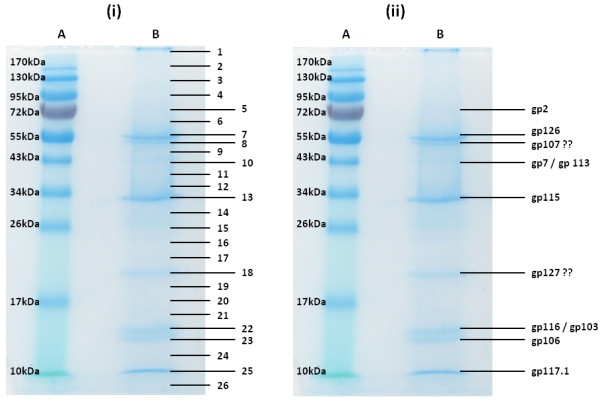
**SDS-PAGE analysis of the purified structural phage proteins (lane B) on a 12% SDS-PAGE separation gel alongside a PageRuler™ prestained protein ladder (Fermentas) (lane A).** The entire lane was cut into numbered slices (i). Subsequently the origin of the visible protein bands were identified by ESI-MS/MS analysis (ii, showing Gps).

Electrospray ionization-MS/MS analysis of the structural phage proteins separated by SDS-PAGE led to the experimental identification of 24 virion proteins with sequence coverage from 8.4 to 85.7% (Table 1/Figure 3). Although only phage proteins with a minimum number of two unique peptides were considered, the identification of gp124 by a single peptide hit was approved based on a reliable proline spectrum [23]. Electrophoretic mobilities of the identified peptides were consistent with their predicted molecular masses, and seven of the nine visible protein bands on the gel could be unambiguously identified (Figure 3). Moreover, traces of the capsid (gp115) and the tail sheath protein (gp126) were identified throughout the gel, which can be explained by aspecific retention and partial degradation of these abundant proteins. 

**Table 1:** Overview of the structural proteins identified by ESI-MS/MS

**Protein number**	**Protein name**	**Protein MW (Da)**	**Max. No. of unique spectra**	**Maxi. sequence coverage (%)**	**Slice in which most abundant**	**Remarks**
gp2	Conserved hypothetical protein	81,838	28	53.9%	5	
gp3	Putative DNA circulation protein	43,673	6	22.6%	9	
gp6	Conserved hypothetical protein	19,283	6	43.3%	20	
gp7	Baseplate protein; phage P2 GpJ homolog	43,379	11	43.9%	9	
gp8	Hypothetical protein	31,431	5	27.3%	13	
gp9	Hypothetical protein	31,400	4	23.2%	13 -14	
gp102	Putative tail fibre	61,504	6	17.7%	4	
gp103	Hypothetical protein	14,237	4	44.7%	22	
gp106	Hypothetical protein	14,221	7	51.7%	23	
gp107	Hypothetical protein	52,046	18	36.9%	1	
gp108	Hypothetical protein	37,504	3	13.5%	11	
gp111	Hypothetical protein	4,295	2	45.2%	25	
gp113	Portal protein	45,459	10	34.2%	9	
gp114	prohead protease	28,590	2	10.5%	18	
gp115	Major capsid protein	52,513	19	46.5%	11 -13 - 14	only 'C-terminal' sequence coverage
gp116	Hypothetical protein	14,261	3	37.9%	20 - 21 - 22	
gp117A	Hypothetical protein	3,205	4	85.7%	25	
gp118	Hypothetical protein	24,820	9	44.3%	14	
gp119	Hypothetical protein	22,041	9	84.7%	17	
gp121	Hypothetical protein	15,986	3	35.0%	19	
gp122	Hypothetical protein	23,047	6	20.5%	15	
gp124	Hypothetical protein	20,253	1	8.4%	18	protein identification probability of 87.70%
gp126	Tail sheath protein	54,066	21	50.5%	7	
gp127	Hypothetical protein	14,475	5	50.7%	18	

For every detected protein the protein name, the predicted molecular weight (Da), the maximum number of unique spectra and sequence coverage (%) is listed. Moreover, the gel slice in which those maximums were observed is given, as well as some additional remarks.

Although the major capsid protein gp115 is clearly the most abundant protein, only peptides of its C-terminus were found. This suggests that the N-terminal part is cleaved off during maturation of the capsid. Indeed, similarity searches indicate that the C-terminal part of gp115 has high similarity with the major capsid protein of the HK97 family and that gp114 is similar to various prohead proteases. As the N-terminal part of the HK97 capsid is cleaved off by a prohead protease encoded by the upstream gene [24,25], the protein band with a molecular weight of approximately 33 kDa refers to the mature major capsid protein.

A final, remarkable finding is the identification of a small, 28 amino acid protein which originally fell below the threshold of gene prediction (i.e. 100 bp). Though the function of this polypeptide is unknown, the high ‘protein identification probability’ of 100% and the coverage of 85.7% confirmed its presence in the phage particle. This proves that proteogenomics, namely the use of proteome analysis to annotate the genome, is a powerful tool to identify missed protein-coding genes and thereby complements genome annotation.

## Discussion

While a number of flagella-specific phages have been isolated – *Salmonella* phage χ, *Caulobacter* phages φCp34 [26], ϕCb13 and ϕCbK [27], and φ6 [28]; *Bacillus* phages AR9, 3NT, PBS1 [29], SP3 [30], and PBP1 [31]; *Proteus* phage PV22 [32]; *Pseudomonas* phage φCTX [33], *Agrobacterium tumefaciens* phages GS2 and GS6 [34]; *Aeromonas hydrophila* phage PM3 [35], and, *Asticcacaulis biprosthecum* φAcS2, and φAcM4 [36] – to the best of our knowledge only χ (Denyes, personal communication) and φCTX [37] have been sequenced. Using the BLASTP feature in BioEdit [38] the products of five 7-7-1 genes (*13, 21, 26, 72* and *102*) possessed homologs in *Salmonella* phage χ. Interestingly, we defined gp102 as a putative tail fibre protein; and, it shows weak sequence similarity from residues 203–300 to a similarly defined protein from phage χ. In view of the quite different tail fibre morphologies observed in phage χ and phage 7-7-1, respectively, the region of similarity may define a general motif involved in phage-flagellum interaction.

Bacteriophage 7-7-1 shows relatively little overall DNA sequence similarity to other phages. At the protein level, CoreGenes revealed eight homologs of BcepB1A proteins, restricted to TerS and a variety of hypothetical proteins. These results indicate that phage 7-7-1 is unique and deserving of recommendation to ICTV as the type phage in a new genus: the 7-7-1-like bacteriophages.

## Materials and methods

### Bacteria and bacteriophages

*Agrobacterium* sp H13-3 (formerly *Rhizobium lupini* H13-3) was isolated from the rhizosphere of *Lupinus luteus*[39]. Phage 7-7-1, which is an isolate from garden compost [40], exclusively infects *Agrobacterium* sp H13-3 [1].

Bacteria were grown in NY medium (8 g nutrient broth, 3 g yeast extract per liter) at 40 rpm in a gyratory shaker at 30 °C. Phage lysates up to 2x10¹¹ PFU per ml were obtained by infection of an exponentially growing culture at OD_650nm_ = 0.1 (8 x 10^7^ CFU per ml) with phage at an MOI of 5 x 10^-3^ followed by threefold dilution with pre-warmed NY and further incubation pending lysis.

### Electron microscopy

Purified phage particles were spread on carbon-coated copper grids, washed once with distilled water and then negatively stained with 4% uranyl acetate, pH 4.8. Microscope magnifications were calibrated with a replica of an optical grating and micrographs were taken with a JEOL 7A (Japan Electron Optics Laboratory Co., Ltd.).

### DNA isolation for sequencing

Phage DNA was isolated by phenol-chloroform extraction [41] and purified by using the Lambda DNA kit of Qiagen (Hilden, Germany). The DNA was subjected to pyrosequencing (454 technology) at the McGill University and Genome Québec Innovation Centre (Montreal, QC, Canada) to 378X coverage.

### Genome annotation

The 7-7-1 sequence was initially subjected to automated annotation using MyRAST (http://blog.theseed.org/servers/presentations/t1/running-a-job-with-the-desktop-rast.html), tRNAScan-SE [42] and ARAGORN [43], following which all open reading frames (ORFs) were confirmed using Kodon (Applied Maths Inc., Austin, TX. USA). The individual proteins were screened against the nonredundant protein databases in NCBI using Batch BLAST (http://greengene.uml.edu/programs/ NCBI_Blast.html). In addition they were screened for conserved motifs using InterProScan [44], Pfam [45], TMHMM v2.0 [46] and Phobius [47].

Putative promoters were identified based upon sequence similarity to the consensus RpoD-specific *E.coli* promoter sequence TTGACA[N15-17]TATAAT while rho-independent terminators were identified using ARNold [48,49] complemented with MFOLD [50].

The genome was submitted to NCBI and accorded accession number JQ312117.

### Comparative genomics

This phage was compared at the DNA and protein levels to other related phages using progressiveMauve [51] and CoreGenes [52,53].

### Proteomics

Structural phage proteins were purified as described by Moak and Molineux [54]. Briefly, a solution of CsCl-purified phage particles (10^11^ PFU) was mixed with methanol and chloroform (1:1:0.75 by volume). After agitation and centrifugation, the upper layer was discarded and an equal volume of methanol was added. The protein pellet obtained by centrifugation at 14, 000 rpm for 6 min, was dried and resuspended in 12.5 mM NH_4_HCO_3_. Subsequently, the heat denatured sample (95 °C, 5 min) was loaded on a 12% SDS-PAGE gel. The Coomassie-stained gel (Simply Blue Safestain; Invitrogen) was cut into slices, which were subjected to trypsin digestion [55]. Peptides were analyzed using electrospray ionization-tandem mass spectrometry (MS/MS) as described previously by Lavigne et al. [56]. The obtained spectra were screened against a database containing all ‘stop-to-stop’ protein sequences in all six frames. Generally, the identification parameters were a ‘protein identification probability’ of at least 99.8% and a ‘best peptide identification probability’ of 95%.

## Abbreviations

BLAST: Basic Local Alignment Search Tool; ESI-MS/MS: electrospray ionization tandem mass spectrometry; Gp:: Gene product; HHpred: Homology detection & structure prediction by HMM-HMM comparison; MOI:: Multiplicity of Infection, ratio of infective phage particles to vulnerable hosts; NY medium: Difco nutrient broth plus yeast extract; PFU:: Plaque Forming Unit, a measure of the number of viable viral particles; SDS-PAGE: denaturing (sodium dodecyl sulfate) polyacrylamide gel electrophoresis; TMHMM: TransMembrane prediction using Hidden Markov Models.

## Competing interests

The authors have no competing interests to disclose.

## Authors’ contributions

AMK performed DNA sequence analyses and sequence annotation, AV, RL and JN analyzed the proteome, PB and RS provided phage DNA, a high-titer lysate, and the fine structure analysis of 7-7-1 particles. All authors contributed to the writing of this manuscript. All authors read and approved the final manuscript.

## Supplementary Material

Additional file 1 **Table S1.** Characteristics of genes and proteins encoded by phage 7-7-1. Click here for file

Additional file 2 **Table S2.** Putative promoters and predicted terminators found in 7-7-1. **Table S3.** Mass spectroscopic analysis of individual gel bands, mzML formatted files in zipped format. Click here for file
