# Supplementary material for: Genome and proteome analysis of 7-7-1, a flagellotropic phage infecting Agrobacterium sp H13-3
Source: Virol J. 2012 May 31;9:102. doi: 10.1186/1743-422X-9-102 (PMC3517404; doi:10.1186/1743-422X-9-102)
Supplement: Additional file 1 — Table S1. Characteristics of genes and proteins encoded by phage 7-7-1. [file 1743-422X-9-102-S1.doc]

Additional File 1. Table S1. Characteristics of genes and proteins encoded by phage 7-7-1

| **Gene** | **Coordinates** | **Strand** | **Length (nt)** | **Protein Mass** | **pI** | **Length (AA)** | **Function** | **Homologs & motifs** | **BLAST expect score** |
| --- | --- | --- | --- | --- | --- | --- | --- | --- | --- |
| 1 | 222..362 | + | 141 | 5187 | 10.0 | 46 | hypothetical protein |  |  |
| 2 | 369..2705 | + | 2337 | 81855 | 9.9 | 778 | conserved structural protein | YP_001111179.1 gp29, bacteriophage membrane protein [Burkholderia phage phiE12-2] | 0.001 |
| 3 | 2710..3906 | + | 1197 | 43692 | 4.9 | 398 | putative tail structural protein | NP_543100.1 hypothetical protein P27p48 [Enterobacteria phage phiP27]; PF07157.6 DNA_circ_N Family | 5.E-07 |
| 4 | 3903..5267 | + | 1365 | 49249 | 6.1 | 454 | putative tail biosynthetic protein | YP_001242396.1 putative Mu-like prophage tail protein [Bradyrhizobium sp. BTAi1]; PF05954.5 Phage_GPD Family | 3.E-12 |
| 5 | 5264..5833 | + | 570 | 20868 | 4.6 | 189 | conserved hypothetical protein | YP_259121.1 baseplate assembly protein V [Pseudomonas fluorescens Pf-5] | 0.01 |
| 6 | 5837..6373 | + | 537 | 19302 | 7.5 | 178 | conserved structural protein | YP_004122139.1 GP46 family protein [Desulfovibrio aespoeensis Aspo-2]; PF07409.6 GP46 Family | 0.002 |
| 7 | 6376..7566 | + | 1191 | 43399 | 4.6 | 396 | baseplate protein; phage P2 GpJ homolog | YP_009443.1 tail protein [Desulfovibrio vulgaris str. Hildenborough]; PF04865.8 Baseplate_J Family | 2.E-08 |
| 8 | 7566..8426 | + | 861 | 31451 | 4.6 | 286 | structural protein |  |  |
| 9 | 8438..9334 | + | 897 | 31418 | 4.3 | 298 | structural protein |  |  |
| 10 | 9331..10152 | + | 822 | 29630 | 9.7 | 273 | conserved hypothetical membrane protein | NP_108221.1 hypothetical protein mlr8035 [Mesorhizobium loti MAFF303099]; one transmembrane domain found using TMHMM & Phobius; PF09374.4 PG_binding_3 Domain & PF05838.6 DUF847 Domain | 4.E-57 |
| 11 | 10149..10352 | + | 204 | 7589 | 8.7 | 67 | conserved hypothetical protein | YP_001313506.1 hypothetical protein Smed_4776 [Sinorhizobium medicae WSM419] | 2.E-11 |
| 12 | 10340..10570 | + | 231 | 8119 | 8.5 | 76 | conserved hypothetical protein | NP_437759.1 hypothetical protein SM_b20921 [Sinorhizobium meliloti 1021] | 2.E-17 |
| 13 | 10729..11205 | - | 477 | 17697 | 6.8 | 158 | conserved hypothetical protein | YP_001418551.1 hypothetical protein Xaut_3666 [Xanthobacter autotrophicus Py2] | 7.E-17 |
| 14 | 11186..11656 | - | 471 | 17456 | 9.3 | 156 | conserved hypothetical protein | ADI19596.1 uncharacterized protein conserved in bacteria [uncultured SAR11cluster bacterium HF0770_37D02] | 5.E-04 |
| 15 | 11653..11961 | - | 309 | 11391 | 5.8 | 102 | conserved hypothetical protein | NP_356864.1 hypothetical protein Atu3758 [Agrobacterium tumefaciens str. C58]; PF10073.3 DUF2312 Family | 6.E-26 |
| 16 | 11958..12266 | - | 309 | 11536 | 5.1 | 102 | hypothetical protein |  |  |
| 17 | 12310..14214 | - | 1905 | 73926 | 7.4 | 634 | putative DNA polymerase | YP_001293433.1 hypothetical protein ORF026 [Pseudomonas phage 73] & YP_001781753.1 phage DNA polymerase [Clostridium botulinum B1 str. Okra] | 5.E-38 |
| 18 | 14214..14378 | - | 165 | 6326 | 6.1 | 54 | hypothetical protein |  |  |
| 19 | 14387..14551 | - | 165 | 6196 | 10.0 | 54 | hypothetical protein |  |  |
| 20 | 14613..15530 | - | 918 | 32019 | 6.1 | 305 | conserved hypothetical protein | YP_024902.1 gp68 [Burkholderia phage BcepB1A] | 6.E-10 |
| 21 | 15549..16640 | - | 1092 | 40867 | 7.1 | 363 | conserved hypothetical protein | YP_024901.1 gp67 [Burkholderia phage BcepB1A]; PF10926.2 DUF2800 Family | 2.E-28 |
| 22 | 16651..17394 | - | 744 | 25716 | 4.6 | 247 | conserved hypothetical protein | YP_024900.1 gp66 [Burkholderia phage BcepB1A] | 0.001 |
| 23 | 17473..19077 | + | 1605 | 59855 | 8.7 | 534 | putative helicase | YP_291195.1 gp65 [Burkholderia phage BcepB1A]; PF00271.25 Helicase_C Family & PF04851.9 ResIII Family | 2.E-84 |
| 24 | 19079..19537 | + | 459 | 17297 | 6.2 | 152 | conserved hypothetical protein | YP_024897.1 gp62 [Burkholderia phage BcepB1A]; PF08774.5 VRR_NUC Domain | 8.E-15 |
| 25 | 19586..19846 | + | 261 | 9697 | 10.1 | 86 | hypothetical protein |  |  |
| 26 | 19849..22194 | + | 2346 | 89497 | 6.4 | 781 | conserved hypothetical protein | NP_102277.1 hypothetical protein mll0485 [Mesorhizobium loti MAFF303099] & YP_024889.1 gp49 [Burkholderia phage BcepB1A] | 2E-65, 3E-26 |
| 27 | 22575..23018 | + | 444 | 16211 | 9.2 | 147 | hypothetical protein |  |  |
| 28 | 23023..23790 | + | 768 | 28209 | 9.0 | 255 | conserved hypothetical protein | YP_002911558.1 hypothetical protein bglu_1g17170 [Burkholderia glumae BGR1]; PF02195.12 ParBc Family & ParB[smart00470], ParB-like nuclease domain & PF08535.4 KorB Domain | 3.E-21 |
| 29 | 23832..24206 | + | 375 | 14538 | 5.2 | 124 | conserved hypothetical protein | YP_001522867.1 hypothetical protein PPLKA1_gp26 [Pseudomonas phage LKA1] | 0.021 |
| 30 | 24221..25336 | + | 1116 | 41671 | 5.2 | 371 | conserved hypothetical protein | Similarity to C-terminus of YP_004517.1 putative cytoplasmic protein [Thermus thermophilus HB27] | 7.E-15 |
| 31 | 25336..25524 | + | 189 | 6916 | 9.7 | 62 | hypothetical membrane protein | 1 TMD |  |
| 32 | 25521..25859 | + | 339 | 12802 | 5.9 | 112 | conserved hypothetical protein | YP_001886460.1 gp91 [Clostridium botulinum B str. Eklund 17B]; PF12961.1 DUF3850 Family | 1.E-08 |
| 33 | 25920..26519 | + | 600 | 22203 | 5.5 | 199 | conserved hypothetical protein | YP_001667953.1 hypothetical protein PputGB1_1714 [Pseudomonas putida GB-1]; PF05014.9 Nuc_deoxyrib_tr Family | 2.E-10 |
| 34 | 26519..26722 | + | 204 | 7717 | 9.5 | 67 | hypothetical protein |  |  |
| 35 | 26719..26913 | + | 195 | 7298 | 8.2 | 64 | conserved hypothetical protein | YP_003358516.1 hypothetical protein [Shigella phage phiSboM-AG3] | 5.E-04 |
| 36 | 26910..27281 | + | 372 | 13188 | 9.6 | 123 | conserved hypothetical protein | YP_498255.1 hypothetical protein Saro_2986 [Novosphingobium aromaticivorans DSM 12444]; PF10686.3 DUF2493 Family | 2.E-22 |
| 37 | 27319..27453 | + | 135 | 5160 | 6.0 | 44 | hypothetical protein |  |  |
| 38 | 27450..27590 | + | 141 | 5186 | 7.9 | 46 | hypothetical protein |  |  |
| 39 | 27583..28065 | + | 483 | 17650 | 6.9 | 160 | conserved hypothetical protein | YP_004302047.1 hypothetical protein SL003B_0316 [Polymorphum gilvum SL003B-26A1] | 2.E-31 |
| 40 | 28062..28487 | + | 426 | 16263 | 5.8 | 141 | hypothetical protein |  |  |
| 41 | 28500..28700 | + | 201 | 7366 | 11.0 | 66 | hypothetical protein |  |  |
| 42 | 28697..28882 | + | 186 | 6761 | 9.9 | 61 | hypothetical protein |  |  |
| 43 | 28879..29220 | + | 342 | 12804 | 9.6 | 113 | hypothetical membrane protein | 1 TMD |  |
| 44 | 29217..29567 | + | 351 | 12974 | 5.3 | 116 | hypothetical protein |  |  |
| 45 | 29564..29752 | + | 189 | 6906 | 5.3 | 62 | hypothetical protein |  |  |
| 46 | 29749..29955 | + | 207 | 7779 | 9.4 | 68 | hypothetical protein |  |  |
| 47 | 29955..30176 | + | 222 | 8251 | 6.2 | 73 | hypothetical protein |  |  |
| 48 | 30227..30640 | + | 414 | 15568 | 9.4 | 137 | hypothetical protein |  |  |
| 49 | 30637..31104 | + | 468 | 17711 | 9.3 | 155 | hypothetical protein |  |  |
| 50 | 31101..31529 | + | 429 | 16237 | 5.7 | 142 | hypothetical protein |  |  |
| 51 | 31526..31951 | + | 426 | 16618 | 9.4 | 141 | hypothetical protein |  |  |
| 52 | 31941..32174 | + | 234 | 8381 | 4.8 | 77 | hypothetical protein |  |  |
| 53 | 32171..32563 | + | 393 | 15305 | 5.2 | 130 | conserved hypothetical protein | YP_001949952.1 hypothetical protein [Ralstonia phage RSL1] | 9.E-11 |
| 54 | 32708..33430 | + | 723 | 27191 | 9.4 | 240 | conserved hypothetical protein | YP_004327556.1 putative uncharacterised protein [Salmonella phage Vi01] | 9.E-23 |
| 55 | 33493..33699 | + | 207 | 7535 | 6.0 | 68 | hypothetical protein |  |  |
| 56 | 33696..33887 | + | 192 | 7176 | 10.3 | 63 | hypothetical protein |  |  |
| 57 | 33887..34123 | + | 237 | 9136 | 9.5 | 78 | hypothetical protein |  |  |
| 58 | 34120..34338 | + | 219 | 8307 | 5.0 | 72 | hypothetical protein |  |  |
| 59 | 34335..34559 | + | 225 | 8118 | 4.6 | 74 | hypothetical protein |  |  |
| 60 | 34556..34882 | + | 327 | 12667 | 8.0 | 108 | hypothetical protein |  |  |
| 61 | 34879..35166 | + | 288 | 11756 | 10.1 | 95 | hypothetical protein |  |  |
| 62 | 35163..35351 | + | 189 | 6910 | 6.0 | 62 | hypothetical protein |  |  |
| 63 | 35348..35638 | + | 291 | 10770 | 8.7 | 96 | hypothetical protein |  |  |
| 64 | 35699..35875 | + | 177 | 6359 | 6.5 | 58 | hypothetical protein |  |  |
| 65 | 35877..36083 | + | 207 | 8112 | 9.6 | 68 | hypothetical protein |  |  |
| 66 | 36080..36394 | + | 315 | 11989 | 6.3 | 104 | hypothetical protein |  |  |
| 67 | 36387..36608 | + | 222 | 8580 | 7.9 | 73 | hypothetical protein |  |  |
| 68 | 36610..36846 | + | 237 | 8784 | 9.0 | 78 | putative transcriptional regulator | YP_001925417.1 XRE family transcriptional regulator [Methylobacterium populi BJ001]; PF01381.16 HTH_3 Domain | 8.E-10 |
| 69 | 36839..37057 | + | 219 | 8264 | 6.3 | 72 | hypothetical protein |  |  |
| 70 | 37044..37277 | + | 234 | 8785 | 10.5 | 77 | hypothetical protein |  |  |
| 71 | 37274..37444 | + | 171 | 6079 | 9.0 | 56 | hypothetical protein |  |  |
| 72 | 37441..37629 | + | 189 | 7436 | 10.1 | 62 | hypothetical protein |  |  |
| 73 | 37854..38426 | + | 573 | 20918 | 9.3 | 190 | conserved hypothetical protein | Similarity to C-terminus of: NP_899614.1 hypothetical protein KVP40.0369 [Vibrio phage KVP40] | 1.E-05 |
| 74 | 38428..38751 | + | 324 | 12230 | 4.2 | 107 | hypothetical protein |  |  |
| 75 | 38748..38912 | + | 165 | 6167 | 8.1 | 54 | hypothetical protein |  |  |
| 76 | 38905..39096 | + | 192 | 7323 | 4.6 | 63 | hypothetical membrane protein | one transmembrane domain found with TMHMM & SPLIT |  |
| 77 | 39156..39356 | + | 201 | 7641 | 8.1 | 66 | hypothetical protein |  |  |
| 78 | 39358..39549 | + | 192 | 6964 | 4.5 | 63 | hypothetical protein |  |  |
| 79 | 39542..39772 | + | 231 | 8883 | 5.3 | 76 | hypothetical protein |  |  |
| 80 | 39765..40136 | + | 372 | 13726 | 7.0 | 123 | hypothetical protein |  |  |
| 81 | 40133..40474 | + | 342 | 12840 | 5.4 | 113 | conserved hypothetical protein | YP_361623.1 hypothetical protein XCVd0064 [Xanthomonas campestris pv. vesicatoria str. 85-10] | 4.E-09 |
| 82 | 40471..41181 | + | 711 | 26972 | 4.9 | 236 | hypothetical protein |  |  |
| 83 | 41979..42782 | + | 804 | 30674 | 9.0 | 267 | conserved hypothetical protein | YP_002435514.1 hypothetical protein DvMF_1092 [Desulfovibrio vulgaris str. 'Miyazaki F'] | 6.E-06 |
| 84 | 42767..43234 | + | 468 | 17645 | 8.8 | 155 | hypothetical protein |  |  |
| 85 | 43244..43894 | + | 651 | 24329 | 4.9 | 216 | hypothetical protein |  |  |
| 86 | 43891..44136 | + | 246 | 8990 | 4.4 | 81 | hypothetical protein |  |  |
| 87 | 44133..44336 | + | 204 | 7871 | 9.5 | 67 | hypothetical protein |  |  |
| 88 | 44333..44695 | + | 363 | 13835 | 9.6 | 120 | conserved hypothetical protein | YP_001101936.1 hypothetical protein SNSL254_pSN254_0057 [Salmonella enterica subsp. enterica serovar Newport str. SL254] | 4.E-07 |
| 89 | 44685..44876 | + | 192 | 7189 | 9.9 | 63 | hypothetical protein |  |  |
| 90 | 44842..45045 | + | 204 | 7823 | 10.2 | 67 | hypothetical protein |  |  |
| 91 | 45052..45723 | + | 672 | 24807 | 9.9 | 223 | putative protein |  |  |
| 92 | 45720..46058 | + | 339 | 12095 | 5.7 | 112 | hypothetical protein |  |  |
| 93 | 46055..46264 | + | 210 | 7632 | 10.2 | 69 | hypothetical protein |  |  |
| 94 | 46275..46754 | + | 480 | 17507 | 6.2 | 159 | conserved hypothetical protein | C-terminus YP_002117621.1 p063 [Rhizobium phage 16-3] | 3.E-05 |
| 95 | 46751..46885 | + | 135 | 4713 | 5.6 | 44 | hypothetical membrane protein | 1 TMD |  |
| 96 | 46882..47445 | + | 564 | 21264 | 7.2 | 187 | conserved hypothetical protein | YP_001758347.1 hypothetical protein Mrad2831_5719 [Methylobacterium radiotolerans JCM 2831] | 3.E-07 |
| 97 | 47518..47769 | + | 252 | 9203 | 4.7 | 83 | hypothetical protein |  |  |
| 98 | 47759..48154 | + | 396 | 14958 | 4.6 | 131 | hypothetical protein |  |  |
| 99 | 48151..48726 | + | 576 | 22378 | 5.3 | 191 | hypothetical protein |  |  |
| 100 | 48780..49307 | + | 528 | 19586 | 5.5 | 175 | terminase, small subunit | YP_612795.1 terminase small subunit [Ruegeria sp. TM1040]; Terminase_2 [pfam03592], Terminase small subunit | 2.E-09 |
| 101 | 49345..49599 | + | 255 | 9366 | 6.6 | 84 | conserved hypothetical membrane protein | YP_223928.1 gp04 [Phage phiJL001]; 1 TMD | 2.E-13 |
| 102 | 49601..51364 | + | 1764 | 61523 | 4.1 | 587 | putative tail fibre | YP_001273684.1 adhesin-like protein [Methanobrevibacter smithii ATCC 35061]; YP_002922323.1 large tail fiber proximal subunit [Enterobacteria phage JSE] shown with PSI-BLAST; PF07602.5 DUF1565 Family | 0.01 |
| 103 | 51386..51811 | + | 426 | 14257 | 5.1 | 141 | structural protein |  |  |
| 104 | 51823..52320 | + | 498 | 17178 | 3.6 | 165 | hypothetical protein |  |  |
| 106 | 52333..52764 | + | 432 | 14240 | 4.3 | 143 | structural protein |  |  |
| 107 | 52786..54294 | + | 1509 | 52065 | 5.0 | 502 | structural protein |  |  |
| 108 | 54296..55318 | + | 1023 | 37523 | 4.4 | 340 | structural protein |  |  |
| 109 | 55312..56739 | + | 1428 | 50190 | 4.9 | 475 | structural protein |  |  |
| 110 | 56772..56981 | + | 210 | 7825 | 6.7 | 69 | hypothetical membrane protein | 1 TMD |  |
| 111 | 56983..57111 | + | 129 | 4314 | 11.8 | 42 | structural protein |  |  |
| 112 | 57113..58297 | + | 1185 | 45555 | 8.5 | 394 | terminase, large subunit | YP_001355936.1 phage terminase, large subunit [Nitratiruptor sp. SB155-2]; Terminase_3 (PF04466), terminase large subunit | 1.E-53 |
| 113 | 58298..59545 | + | 1248 | 45477 | 6.0 | 415 | portal protein | YP_001937155.1 hypothetical protein OTT_0463 [Orientia tsutsugamushi str. Ikeda]; Phage_portal (PF04860), phage portal protein | 6.E-12 |
| 114 | 59532..60305 | + | 774 | 28609 | 5.0 | 257 | prohead protease | YP_001627270.1 HK97 family phage prohead protease [Brucella suis ATCC 23445]; Peptidase_U35 (PF04586) Caudovirus prohead protease | 1.E-11 |
| 115 | 60326..61735 | + | 1410 | 52531 | 5.1 | 469 | major capsid protein | YP_916678.1 HK97 family phage major capsid protein [Paracoccus denitrificans PD1222]; Phage_capsid (PF05065), phage capsid family | 8.E-04 |
| 116 | 61802..62209 | + | 408 | 14280 | 4.2 | 135 | structural protein |  |  |
| 117 | 62548..63096 | + | 549 | 19693 | 4.6 | 182 | hypothetical protein |  |  |
| 117A | 63133..63219 | + | 87 | 3205 | 9.6 | 28 | structural protein |  |  |
| 118 | 63283..63975 | + | 693 | 24839 | 4.6 | 230 | structural protein |  |  |
| 119 | 63972..64580 | + | 609 | 22060 | 8.4 | 202 | structural protein |  |  |
| 120 | 64577..64705 | + | 129 | 5227 | 8.7 | 42 | hypothetical protein |  |  |
| 121 | 64702..65124 | + | 423 | 16005 | 7.8 | 140 | structural protein |  |  |
| 122 | 65136..65798 | + | 663 | 23066 | 4.6 | 220 | structural protein |  |  |
| 123 | 65788..66204 | + | 417 | 16146 | 11.0 | 138 | hypothetical protein |  |  |
| 124 | 66189..66728 | + | 540 | 20271 | 4.8 | 179 | structural protein |  |  |
| 125 | 66732..66938 | + | 207 | 7086 | 9.5 | 68 | hypothetical protein |  |  |
| 126 | 66945..68456 | + | 1512 | 54086 | 4.6 | 503 | tail sheath protein | YP_192735.1 bacteriophage tail sheath protein [Gluconobacter oxydans 621H]; PF04984.8 Phage_sheath_1 Family | 2.E-14 |
| 127 | 68472..68882 | + | 411 | 14494 | 4.4 | 136 | structural protein |  |  |
