# Supplementary material for: Genome and proteome analysis of 7-7-1, a flagellotropic phage infecting Agrobacterium sp H13-3
Source: Virol J. 2012 May 31;9:102. doi: 10.1186/1743-422X-9-102 (PMC3517404; doi:10.1186/1743-422X-9-102)
Supplement: Additional file 2 — Table S2. Putative promoters and predicted terminators found in 7-7-1. Table S3. Mass spectroscopic analysis of individual gel bands, mzML formatted files in zipped format. [file 1743-422X-9-102-S2.doc]

Additional File 2. Table S2– Putative promoters and predicted terminators found in 7-7-1.

| Promoter | Position | Sequence |
| --- | --- | --- |
| P_22_ | 17457..17430 | TTGACAcatatttgcataaTGagcTAAT |
| P_23_ | 17417..17442 | TTGACgcaaaacgattagctcATtAT |
| P_27_ | 22399..22426 | TTGACgtgttaacacaaattcgcATAAT |
| P_37_ | 27265..27291 | TTGACAactacaaatagtttgTATAAg |
| P_48_ | 30173..30199 | TTGACtgatacaaatagattgTATAAT |

The capital letters in the sequence indicate bases with are identical to the conserved residues

In the -35 and -10 regions of the consensus promoter TTGACA(N15-17)TATAAT

| Terminator | Position | Sequence | ΔG (kcal/mol) |
| --- | --- | --- | --- |
| t_20_ | 14592..14563 | **ggcggggga**ttatc**tcccgcc**gttttttat | -17.5 |
| t_53_ | 32596..32636 | **ccccacgata**ccggcccgtgaaccggaaaaa**tatcgtgggg** | -21.4 |
| t_72_ | 37735..37772 | **ggggcg**ggttaacaggtggtgctggaccataa**cgcccc** | -15.8 |
| t_82_ | 41210..41242 | **aggggct**a**gg**gtaaca**ccggcccct**tttatttt | -15.0 |
| t_117A_ | 63240..63263 | **cgggcgg**ttaaaa**ccgcccg**tttt | -15.6 |

N.B. Lower case numbering denotes the gene next to the promoter or terminator; the bold underlined

Nucleotides for the stem structure of the predicted terminators
